# Cerebrovascular Autoregulation in Preterm Infants Using Heart Rate or Blood Pressure: A Pilot Study

**DOI:** 10.3390/children11070765

**Published:** 2024-06-24

**Authors:** Bineta E. Lahr, Celina L. Brunsch, Riksta Dikkers, Arend F. Bos, Elisabeth M. W. Kooi

**Affiliations:** 1 Department of Neonatology, Beatrix Children’s Hospital, University Medical Center Groningen, 9713 GZ Groningen, The Netherlands; b.e.lahr@umcg.nl (B.E.L.); c.l.brunsch@umcg.nl (C.L.B.);; 2 Department of Pediatric Radiology, Beatrix Children’s Hospital, University Medical Center of Groningen, 9713 GZ Groningen, The Netherlands

**Keywords:** neonatology, cerebrovascular circulation, hemodynamics, arterial pressure, heart rate, brain injury, near-infrared spectroscopy

## Abstract

Background: Cerebrovascular autoregulation (CAR) is often impaired in preterm infants but requires invasive mean arterial blood pressure (MABP) measurements for continuous assessment. We aimed to assess whether using heart rate (HR) results in different CAR assessment compared with using MABP. Methods: We compared CAR (moving window correlation-coefficient with cerebral oxygenation saturation (r_c_SO_2_)), and percentage of time with impaired CAR (%timeCARi) calculated by either HR (TOHRx, tissue oxygenation heart rate reactivity index) or MABP (COx, cerebral oximetry index) during the first 72 h after birth, and its association with short-term cerebral injury. Results: We included 32 infants, median gestational age of 25 + 5/7 weeks (interquartile range 24 + 6/7–27 + 5/7). COx and TOHRx correlation coefficients (cc) were significantly different in the first two days after birth (individual means ranging from 0.02 to 0.07 and −0.05 to 0.01). %TimeCARi using MABP (cc cut-off 0.3), was higher on day 1 (26.1% vs. 17.7%) and day 3 (23.4% vs. 16.9%) compared with HR (cc cutoff −0.3). During 65.7–69.6% of the time, both methods indicated impaired CAR simultaneously. The aforementioned calculations were not associated with early cerebral injury. Conclusions: In conclusion, HR and MABP do not seem interchangeable when assessing CAR in preterm infants.

## 1. Introduction

Cerebrovascular autoregulation (CAR), the ability of the brain’s vasculature to maintain stable cerebral blood flow (CBF) regardless of fluctuating cerebral perfusion pressures (CPP), is often impaired in preterm infants [1]. CAR is mainly conceived by cerebral vasoconstriction and vasodilatation. Due to the limited capacity of CAR, the CBF will decrease when CPP decreases below a lower threshold and increase when CPP rises above an upper threshold [1,2]. In preterm infants, inadequate CAR may be an important pathophysiological mechanism causing brain injury, because of the immature vasculature in the germinal matrix and periventricular white matter in preterm infants [1,2,3]. Both hypoperfusion and hyperperfusion can cause significant perinatal injuries to the central nervous system, such as germinal matrix-intraventricular hemorrhage (IVH) on the one hand, and periventricular leukomalacia (PVL) on the other [3,4,5]. IVH is the most commonly diagnosed brain injury in premature newborns and 80–90% of IVH occur during the transitional period, the first 72 h after birth, which is often characterized by hemodynamic instability [4,6]. PVL, resulting from perinatal ischemic injury, is frequently seen in combination with IVH [3]. Both may lead to potentially severe neonatal complications and adverse lifelong neurodevelopmental outcomes, such as cerebral palsy and developmental delay [3,7,8]. In early life, factors such as lower birth weight (BW), hemodynamically significant patent ductus arteriosus (hsPDA), higher pCO_2_, and being small for gestational age (SGA) seem associated with impaired CAR [9,10,11]. Impaired CAR is also associated with increased infant mortality [12].

CAR can be assessed by determining the interaction between CPP and CBF, with a positive correlation between the two implying a CPP-dependent CBF, suggesting impaired CAR [13]. In preterm infants, the assessment of CAR is based on surrogates for both CPP and CBF because both cannot be measured directly in these vulnerable infants [9]. Near-infrared spectroscopy (NIRS) has been used for a continuous and non-invasive assessment of cerebral oxygen saturation (r_c_SO_2_), which reflects the balance between oxygen supply to the brain and its oxygen use. It can act as a surrogate for CBF assuming stable metabolic demand [9,13]. The mean arterial blood pressure (MABP) minus the intracranial pressure (ICP) defines the CPP. Since the ICP is assumed to be low in neonates and cannot be measured directly, the MABP is often used as a surrogate for CPP [14,15]. However, the blood pressure is not identical to CPP during the transitional period because of the presence of intracardiac and extracardiac shunts, resulting in diverse blood pressure values at various locations [9]. In addition, preterm infants are less able to increase stroke volume, but instead achieve this by increasing their heart rate (HR). Assessment of dynamic autoregulation relies on spontaneous fluctuations of physiological variables. In preterm infants, the invasive measurement of MABP is not always feasible. Conversely, their HR is easily assessed, and is more variable [16]. Since HR can be measured non-invasively, determining CAR based on HR would be a more feasible alternative. Despite numerous studies about CAR ability in premature infants [1,2,3,4,10,16,17,18], little research has been done into assessing HR as a surrogate for CPP [16]. To the best of our knowledge, HR has never been compared to MABP in CAR assessment with regards to their association with short-term neurodevelopmental outcomes.

In this pilot study, we first aimed to compare, in very preterm infants, the absolute correlation coefficients (cc) of regional cerebral oxygen saturation (r_c_SO_2_) with MABP and with HR. Second, we evaluated whether the percentage of time with impaired CAR (%timeCARi) differed between both methods, during the first 72 h after birth. Third, we assessed if these two methods to assess CAR differed in association with short-term cerebral injury (IVH/PVL) on cranial ultrasonography (cUS). Based on a previous observation [16], we hypothesized that HR and MABP are similarly useful for assessing CAR in preterm infants, but that HR is a better surrogate for CPP than the MABP when identifying adverse clinical outcomes.

## 2. Materials and Methods

### 2.1. Patient Population and Data Collection

We conducted a single-center observational study in a selection of infants included in two ongoing prospective cohort trials [19], studying risk factors for necrotizing enterocolitis and adverse neurodevelopmental outcomes. All infants who were born below 30 weeks of gestation and/or with a birthweight < 1000 g were included in these prospective cohort trials. Infants were excluded in case of severe congenital or chromosomal abnormalities. For the current study, we included participating infants when they were born between 1 May 2020 and 28 February 2023. Additional inclusion criteria were the availability of measurements of r_c_SO_2_, HR and MABP via an indwelling catheter for a minimum of six hours during the first 24 h and continuously ongoing during the next 48 h after birth. Measurements of r_c_SO_2_ using NIRS and HR are part of the standard care for neonates in our NICU. MABP was only assessed when an indwelling arterial catheter was inserted for clinical reasons, either centrally via the umbilical artery or peripherally at either extremity, as indicated by the attending physician. Parental written informed consent was obtained in all cases. This study was approved by the medical ethical committee of the University Medical Center Groningen (METc 2019/235 and METc 2013/263).

### 2.2. Difference in Cerebrovascular Autoregulation Assessment Based on MABP HR

To assess COx (cerebral oximetry index) and TOHRx (tissue oxygenation heart rate reactivity index), we calculated Pearson’s cc between MABP and r_c_SO_2_ or HR and r_c_SO_2_, respectively, using a 10-min moving window with maximal overlap for every paired sample, as explained in more detail elsewhere [12,15,17,20]. We used the INVOS 5100C Regional Oximeter (Medtronic, Dublin, Ireland) with neonatal sensors (Medtronic, Dublin, Ireland) to provide real-time changes in r_c_SO_2_. All data, including vital data such as MABP, HR, and oxygen saturation (SpO_2_), were transferred automatically to an offline database, with a sampling frequency of 0.2 Hz. We determined CAR based on either MABP or HR during six-h monitoring episodes per day in the first 72 h after birth, using r_c_SO_2_ as surrogate for CBF [15,16]. The measurement periods were selected based on continuously available data of r_c_SO_2_ and MABP per 24-h-period. We allowed fluctuations in both values and manually removed conspicuous artifacts of r_c_SO_2_, including a sudden extensive non-physiologic increase or decrease of r_c_SO_2_ values, indicating incorrect measurement. These disturbances could be related to movement, medication, or nursing care.

We chose our cc cut-off value for impaired CAR based on a literature review, which presented cut-offs ranging between 0 and 0.5 when using MABP [9]. Earlier, a positive correlation between left ventricular output and CBF has been reported [16]. In case of a low cardiac output, HR increases as a compensatory mechanism to increase the CO, until its maximum capacity at a certain higher HR, after which the HR can no longer increase [21]. Thus, negative cc might be more suitable for impaired CAR based on HR. However, previous studies have used positive cc to define impaired CAR based on HR [4,18]. For this reason, we decided to present both positive (0.3) and negative (−0.3) cc cut-off values for impaired CAR based on HR.

### 2.3. Short-Term Cerebral Injury

CUS was used to determine cerebral injury in our study population. All examinations were performed by trained pediatric radiologists with an Arietta 850, Hitachi ultrasound machine with a convex 4–8 MHz transducer through the anterior fontanel and mastoid fontanel to detect IVH and PVL development. The cUS were carried out as soon as possible after birth, but at least within three days after birth, and were repeated after one week according to protocol. A specialized pediatric radiologist (RD) re-evaluated the ultrasound images of all patients. The severity of IVH was classified according to Papile et al. [22]. However, we described periventricular hemorrhage as the presence of periventricular hemorrhagic infarction (PVHI) instead of grade IV hemorrhage [23]. For PVL detection, we used the PVL classification system described by De Vries et al. [24]. We defined ‘short-term cerebral injury on cUS’ in case of severe hemorrhage (IVH grade III or presence of PVHI), worsening of IVH in any grade on any site, or development of PVL in the first 10 days after birth. Since we focused on evaluating CAR during the transitional period, we chose to examine the development of short-term cerebral injury closest to our measurement periods.

We collected clinical data to detect potential other risk factors for cerebral injury from the patients’ files: gestational age (GA), 5 min Apgar score, SGA (defined as a birthweight of <10th percentile on the Dutch Hoftiezer growth curves), hsPDA based on echocardiographic findings, NEC (necrotizing enterocolitis), EONS (early onset of neonatal sepsis confirmed with a positive blood cult), sedatives, inotropes, pCO_2_, and mechanical ventilation [25,26,27].

### 2.4. Statistical Analysis

We used the Statistical Package for the Social Sciences (SPSS) 28.0 software for the statistical analysis. We checked for normality of the data to decide on parametric or non-parametric tests. We performed a paired *t*-test to test differences between absolute COx and TOHRx cc per day. We performed a paired *t*-test to test differences in mean %timeCARi impaired CAR using MABP (COx > 0.3) or HR (TOHRx > 0.3 or <−0.3). We used Pearson’s correlation tests to identify correlations between COx and TOHRx for every day. We performed *t*-tests to test differences between COx and TOHRx and %timeCARi impaired CAR using COx and TOHRx, between infants with and without short-term cerebral injury on cUS. We then performed a univariable logistic model to detect the clinical determinants of cerebral injury on cUS. Next, we built four multivariable models with all variables that were associated with cerebral injury with *p* < 0.1 from the univariable analyses. In this exploratory pilot study, multiple testing was not corrected. *p*-values of <0.05 (two-tailed) were considered statistically significant.

## 3. Results

### 3.1. Patient Characteristics

**Figure 1:**
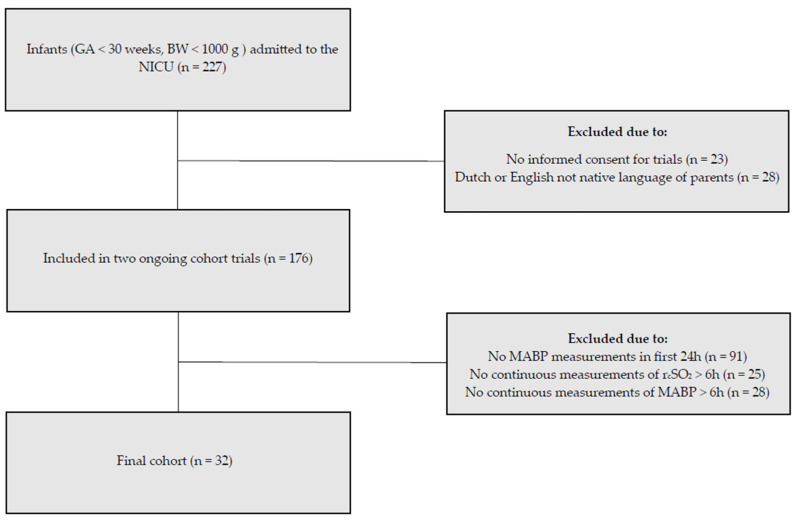
Inclusion flow diagram. Of the 227 neonates admitted to our neonatal intensive care unit, 32 met the inclusion criteria for this substudy.

During our study period, 176 infants were included in the ongoing cohort studies. For the present study, we included the 32 infants with available MABP and r_c_SO_2_ measurements (Figure 1). The demographics, comorbidities, and outcomes are summarized in Table 1. Median GA was 25 ± 5/7 weeks and days (interquartile range 24 + 6/7–27 + 5/7), and the mean BW was 902 ± 244 g. In the first 72 h after birth, 63% on day one, 53% on day two, and 47% on day three of the infants in our study required mechanical ventilation (Table 2). 94% Of the infants needed surfactant replacement during the study period.

### 3.2. Cerebrovascular Autoregulation Assessment Using Either MABP or HR

We found positive mean COx cc (individual means ranging from 0.02 to 0.07) and negative mean TOHRx cc (individual means ranging from −0.05 to −0.01) on all 3 days. COx and TOHRx cc differed on days one and two (day one *p* = 0.003, day two *p* = 0.004, day three *p* = 0.398) (Table 3). On day one, COx correlated negatively with TOHRx (*r* = −0.38, *p* = 0.033). On day two and three, *r* was <0.1 (*p* = 0.998), and −0.30 (*p* = 0.125), respectively.

When comparing both methods for determining impaired CAR using TOHRx with cc cut-off < −0.3 and COx using cc cut-off of 0.3, we found a mean agreement of 69.6 ± 12.5% on day one, 65.7 ± 12.3% on day two, and 69.4 ± 12.1% on day three. Using TOHRx cc > 0.3 as cut off for impaired CAR, compared with COx, we found a mean agreement of 67.6 + 17.3% on day 1, 72.1 ± 11.9% on day two, and 70.1 ± 12.7% on day three, respectively (Table 3).

**Figure 2:**
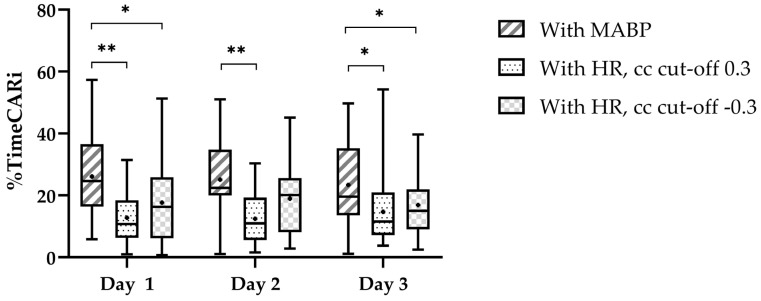
Percentage of time with impaired cerebrovascular autoregulation per day. The central line in the boxplot is the median, the whiskers represent the range and the box margins are the 25th and 75th percentiles. The black dot in the boxplot represents the mean. The significant pairwise comparisons (paired *t*-tests) are highlighted as follows: * *p* < 0.05 and ** *p* < 0.001.

We found a higher %timeCARi using COx on all three days (Figure 2). Pearson’s cc for the correlation between %timeCARi using COx and TOHRx with cc cut-off > 0.3 were *r* = −0.22 (*p* = 0.218) on day one, *r* = 0.25 (*p* = 0.236) on day two, and *r* = −0.09 (*p* = 0.640), on day three. Using TOHRx with cc cut-off < −0.3 these were *r* = 0.35 (*p* = 0.052) on day one, *r* = 0.08 (*p* = 0.713) on day two, and *r* = 0.31 (*p* = 0.113), on day three.

### 3.3. Impaired Cerebrovascular Autoregulation and Short-Term Cerebral Injury

Short-term cerebral injury on cUS as defined for this study, was observed in 20/32 neonates (62.5%). Bilateral IVH (any grade) and PVL were observed in respectively 25/32 (78.1%) and 10/32 (31.3%) neonates. Worsening of IVH in any grade one week after birth was observed 13/32 (40.6%) neonates (Table 4).

We found no significant difference in the mean COx cc between neonates with cerebral injury on cUS and those without. Similarly, no significant differences were found in the mean TOHRx cc between neonates with cerebral injury and those without (Table 5). The mean %timeCARi using MABP or HR did not differ significantly between neonates with short-term cerebral injury one week after birth and those without (Table 5). Subgroups with impaired IVH or PVL one week after birth showed no significant differences %timeCARi cerebral injury on cUS.

On day two, we found a significantly more negative mean TOHRx cc in neonates with any stage IVH on cUS one week after birth (−0.07 + 0.109) versus those without any IVH (0.02 + 0.111, *p* = 0.016) (Table S1a).

Regression analyses revealed that TOHRx, COx, and %timeCARi using both HR and MABP were not associated with cerebral injury on cUS. Neonates who were mechanically ventilated had a significantly higher risk of developing cerebral injury on cUS compared to those who did not on day three (Tables S2 and S3).

## 4. Discussion

In this prospective observational study investigating the assessment of CAR using HR or MABP, we found mainly positive COx cc and negative TOHRx cc and higher %timeCARi CAR using MABP during the daily 6-h monitoring episodes in very preterm infants within the first 72 h after birth. When using COx compared to TOHRx we found that, assuming cc cut-off values of respectively 0.3 and −0.3, both methods were in agreement regarding impaired CAR during less than 73% of the time. We found no association between either COx or TOHRx and cerebral injury on cUS as defined for the purpose of this study. However, more negative TOHRx cc were associated with presence of IVH one week after birth.

Mitra et al. were the first to introduce HR as a potential alternative for MABP to utilize in CAR calculations. They found more positive TOHRx cc in infants with worse clinical performance and higher risk of developing grade 3 IVH [16], which contradicts our findings of more negative TOHRx in infants with IVH one week after birth. We speculate that when an increasing HR reaches its maximum capacity, there is an inability to further increase cardiac output. Our study sample, biased toward infants with a clinical need for indwelling catheters for MABP measurements, potentially comprising more severely ill neonates, might explain the increased incidence of grade 3 IVH compared to the population studied by Mitra et al. [16]. As we expected, more negative TOHRx cc in our study population were observed. Simultaneous COx cc were mainly positive in our study, representing impaired CAR, thus supporting our hypothesis [9]. Cimatti et al. also found higher TOHRx cc before IVH detection during the transitional period in preterm infants, but non-invasive MABP monitoring was not feasible, which might have caused selection bias toward less severely ill neonates [4]. However, in instances where HR has not yet reached its maximum capacity to increase cardiac output, and a decrease in CO results in increasing HR, higher TOHRx cc could possibly be observed as is seen in previous studies [4,16,18].

We now added %timeCARi and found impaired CAR for about 25% of the time utilizing MABP and about 18% utilizing a negative cc cut-off for TOHRx, and even lower utilizing a positive cc cut-off. In our study an agreement between impaired CAR using MABP and both measurement methods using HR appeared to be approximately two-thirds, which renders one-third disagreement between both methods. The consistent numbers of agreement observed for both cc cut-off values of TOHRx may be attributed to the mean TOHRx possibly falling within the range of normal CAR during our six-h monitoring periods. Importantly, by utilizing surrogate markers for CPP and CBF, and thus introducing certain imprecisions, we chose our cut-off based on a literature review, which reported cut-offs ranging between 0.3 and 0.5 when using MABP [9]. The chosen cc cut-off 0.3 for COx and 0.3 or −0.3 for TOHRx to define impaired CAR, rather than just using zero, needs further research [9,15,16,17,20,28]. Different approaches to assess %timeCARi make it difficult to compare and generalize results [29]. The fact that we found relatively high %timeCARi compared to earlier research [17,30,31], suggests that using a cc cut-off of 0.3 reduces erroneous overinterpretation of impaired CAR, which we could not substantiate when looking at short-term cerebral injury.

We could not confirm a relation between COx, TOHRx, and %timeCARi and cerebral injury on cUS. For this study we chose to investigate cUS abnormalities one week after birth, because a golden standard outcome measure for impaired CAR is lacking [29]. Therefore, the definition of short-term cerebral injury on cUS that we composed containing both IVH and PVL, may not validly represent impaired CAR during the first days after birth, for example because (timing of) cranial ultrasound is not sensitive enough to detect subtle cerebral injury. We do believe however, that in order to assess CAR as a dynamic process, it is relevant to measure potential cerebral injury from impaired CAR in both directions simultaneously, i.e., both ischemic (PVL) and hemorrhagic lesions (IVH) [3,4,5]. For this reason, we decided to use a combined outcome measure of severe or worsening IVH, PVHI, and PVL. Also, due to our relatively small sample size, we opted to form moderately sized groups for our outcomes. In addition, despite the hemodynamic predisposition in preterm infants, the etiopathogenesis of cerebral injury is multifactorial, and other causes beyond impaired CAR may contribute [32]. This suggests that our definitions of impaired CAR might not consistently align directly with cerebral injury in our study.

We did find more negative TOHRx cc in neonates with IVH in any grade on cUS one week after birth. Potentially, IVH is a more pronounced consequence of impaired CAR than PVL, which could be explained by the fact that disturbance in CBF and fragility of the germinal matrix during the transitional period contribute to the development of IVH after birth [33]. Evolution of PVL may already occur in the late antenatal period, which would explain why we did not find a relationship between the presence of PVL one week after birth and CAR, as impaired CAR would have less impact on the development of PVL during the early postnatal period [34]. Furthermore, HR may be a more valid alternative to measure CAR non-invasively as MABP is affected by intra- and extracardiac shunts and does not necessarily represent CPP properly. Of note, the association we found between TOHRx and IVH may be incidental, as we chose not to correct for multiple testing.

Our study has several strengths. To our knowledge, this is the first study that compared using HR with using MABP for the assessment of CAR, including calculating %timeCARi, in relation to short-term cerebral injury on cUS. This has led to the suggestion that when using HR as surrogate for CPP, negative correlations might represent impaired CAR, and both methods are not interchangeable. These findings contribute to unraveling the complexity of assessing CAR in relation to cerebral injury in preterm infants. We also recognize several limitations to our study. The first being the relatively small sample size, though not smaller than previously reported samples, and the usage of only early outcome measures [4,16]. Due to the exploratory nature of this pilot study, an a-priori sample size calculation was not performed, and we chose not to correct for multiple testing. A second limitation concerns the inclusion of our subjects. This was dependent on available MABP measurements with an indwelling catheter, leading to selection bias towards more severely ill neonates. In our NICU, arterial catheters are inserted only in the sickest patients, who are more likely to be hemodynamically unstable [12]. That may have led to an overestimation of impaired CAR, although previously we did not confirm an association between level of illness and impaired CAR [35]. Lastly, a potential selection bias of the chosen measurement periods may have occurred, since we manually removed artifacts.

## 5. Conclusions

In conclusion, MABP and HR agree for only 66–72% as input parameters for CAR assessment. Therefore appear not interchangeable. We found mainly positive COx and negative TOHRx, and longer time with impaired CAR when using COx, compared to when using TOHRx. Both COx and TOHRx were not significantly associated with short-term cerebral injury on cUS one week after birth. In absence of a golden standard outcome measure for impaired CAR, HR is not a better surrogate for CPP than the MABP when identifying adverse clinical outcomes based on our definition of short-term cerebral injury. More research is needed into the clinical use of HR to identify and treat impaired CAR as a risk factor for short-term cerebral injury, especially considering the need for alternatives to MABP. 

## Figures and Tables

**Table 1:** Demographics and clinical characteristics of the study cohort (*n* = 32).

Clinical Characteristics	N (%) or Mean ± SD or Median (Interquartile Range)		
Gestational age (weeks + days)	25 + 5/7 (24 + 6/7–27 + 5/7)		
Birth weight (g)	902 ± 244		
Male	16 (50%)		
Multiple pregnancy	14 (43.8%)		
Head circumference (cm)	23.4 ± 1.7		
Apgar scores at 5 min	7 (5–8)		
SGA	4 (12.5%)		
HsPDA	15 (46.9%)		
NEC	7 (21.9%)		
EONS	2 (6.3%)		
	Day 1	Day 2	Day 3
Sedatives	9 (28.1%)	11 (34.4%)	8 (25%)
Inotropes	1 (3.1%)	4 (12.5%)	2 (6.3%)
Mortality during NICU admission	4 (12.5%)		

Data are presented as number (percentage) and either mean ± standard deviation or median (interquartile range). SGA, small for gestational age according to birthweight of <10th percentile [25]; hsPDA; NEC, necrotizing enterocolitis; EONS, early onset of neonatal sepsis confirmed with a positive blood cult; hsPDA, hemodynamically significant patent ductus arteriosus; NICU, neonatal intensive care unit.

**Table 2:** Hemodynamic measures per day.

Measurement Variables	Day 1 (*n* = 32)	Day 2 (*n* = 24)	Day 3 (*n* = 28)
MABP (mmHg)	34 ± 4	37 ± 5	38 ± 6
HR (bpm)	150 ± 2	155 ± 5	157 ± 10
R_c_SO_2_ (%)	79 ± 7	80 ± 7	78 ± 7
SpO_2_ (%)	94 ± 3	94 ± 2	93 ± 2
	(*n* = 32)	(*n* = 32)	(*n* = 32)
Mechanical ventilation	20 (62.5%)	17 (53.1%)	15 (46.9%)
Peak FiO_2_	0.46 [0.32–0.69]	0.32 [0.21–0.66]	0.35 [0.25–0.50]
	(*n* = 30)	(*n* = 25)	(*n* = 28)
PCO_2_ (kPa)	5.3 ± 1.1	5.6 ± 1.1	5.9 ± 1.0

Data are presented as number (percentage) and mean ± standard deviation or as median (interquartile range). MABP, mean arterial blood pressure; HR, heart rate; r_c_SO_2_, cerebral tissue oxygenation saturation; SpO_2_, oxygen saturation; FiO_2_, fraction of inspired oxygen; pCO_2_, partial pressure of carbon dioxide. Numbers vary daily since CAR measurements were not available for all infants on all days in this study.

**Table 3:** Cerebrovascular autoregulation measures per day. Primary endpoint: %timeCARi.

Measurement Variables	Day 1 (*n* = 32)	Day 2 (*n* = 24)	Day 3 (*n* = 28)
Mean COx	0.07 ± 0.11 *	0.06 ± 0.12 *	0.02 ± 0.15
Mean TOHRx	−0.03 ± 0.11	−0.05 ± 0.10	−0.01 ± 0.11
Agreement on impaired CAR (%)			
TOHRx cc cut-off 0.3	67.6 ± 17.3	72.1 ± 11.9	70.1 ± 12.7
TOHRx cc cut-off −0.3	69.6 ± 12.5	65.7 ± 12.3	69.4 ± 12.1

Data are presented as mean ± standard deviation. COx, cerebral oximetry index; TOHRx, tissue oxygenation heart rate reactivity; CAR, cerebrovascular autoregulation; cc, correlation coefficient. Numbers vary daily since CAR measurements were not available for all infants on all days in this study. * *p*-value < 0.05 when compared to using TOHRx.

**Table 4:** Sonographic characteristics on the first cUS after birth or cUS 7–10 days after birth (*n* = 32).

Sonographic Characteristics	First cUS on Day 1–3		cUS on Day 7–10	
Cerebral injury on cUS as defined			20 (62.5%)	
IVH	20 (62.5%)		25 (78.1%)	
Side				
Right-sided	5 (25%)		3 (12%)	
Left-sided	1 (5%)		2 (8%)	
Bilateral	14 (70%)		20 (80%)	
Grade	Right side	Left side	Right side	Left side
I	12 (63.2%)	8 (53.3%)	10 (43.5%)	13 (59.1%)
II	3 (15.8%)	4 26.7%)	4 (17.4%)	4 (18.2%)
III	4 (21.1%)	3 (20.0%)	9 (39.1%)	5 (21.9%)
Worsening of IVH			13 (40.6%)	
PVHI	5 (15.6%)		7 (24.1%)	
PVL			10 (31.3%)	
Side				
Bilateral			10 (100%)	
Grade				
I			10 (100%)	

Data are presented as number (percentage). CUS, cranial ultrasonography; IVH, germinal matrix-intraventricular hemorrhage according to Papile (modified) [22,23]; PVHI, periventricular hemorrhagic infarction; PVL, periventricular leukomalacia according to de Vries et al. [24].

**Table 5:** Cerebrovascular autoregulation and its relation to cerebral injury on cUS per day.

Day	Measurement Variable	No Cerebral Injury on cUS (*n* = 12)	Cerebral Injury on cUS (*n* = 20)	*p*-Value
1	R_c_SO_2_ (%)	80.9 ± 6.4	77.0 ± 6.4	0.111
	COx	0.09 ± 0.10	0.07 ± 0.12	0.589
	TOHRx	−0.03 ± 0.12	−0.03 ± 0.11	0.914
	%TimeCARi with MABP (%)	27.0 ± 13.8	25.6 ± 13.1	0.774
	%TimeCARi with HR (%)			
	TOHRx cc cut-off 0.3	13.7 ± 8.1	12.2 ± 8.6	0.631
	TOHRx cc cut-off −0.3	18.2 ± 13.3	17.4 ± 13.3	0.884
2		(*n* = 7)	(*n* = 17)	
	R_c_SO_2_ (%)	79.7 ± 8.8	79.7 ± 6.4	0.494
	COx	0.03 ± 0.12	0.07 ± 0.13	0.304
	TOHRx	0.00 ± 0.07	−0.07 ± 0.11	0.081
	%TimeCARi with MABP (%)	21.5 ± 8.3	26.6 ± 13.3	0.274
	%TimeCARi with HR (%)			
	TOHRx cc cut-off 0.3	14.6 ± 7.1	11.5 ± 8.9	0.381
	TOHRx cc cut-off −0.3	13.2 ± 10.2	21.3 ± 12.1	0.118
3		(*n* = 10)	(*n* = 18)	
	R_c_SO_2_ (%)	79.4 ± 4.9	77.4 ± 8.1	0.714
	COx	0.06 ± 0.16	0.00 ± 0.15	0.296
	TOHRx	−0.01 ± 0.10	−0.02 ± 0.11	0.751
	%TimeCARi with MABP (%)	26.7 ± 15.6	21.6 ± 11.5	0.332
	%TimeCARi with HR			
	TOHRx cc cut-off 0.3	15.7 ± 9.2	14.1 ± 12.0	0.731
	TOHRx cc cut-off −0.3	16.6 ± 9.4	17.4 ± 10.7	0.928

Data are presented as mean ± standard deviation. CUS, cranial ultrasonography; r_c_SO_2_, cerebral tissue oxygenation saturation; COx, cerebral oximetry index; TOHRx, tissue oxygenation heart rate reactivity index; %timeCARi, percentage of time with impaired cerebrovascular autoregulation; MABP, mean arterial blood pressure; HR, heart rate; cc, correlation coefficient. Numbers vary daily since CAR measurements were not available for all infants on all days in this study.

## Data Availability

The datasets analyzed during the current study are available from the corresponding author on reasonable request.

## Supplementary Materials

The following supporting information can be downloaded at: https://www.mdpi.com/article/10.3390/children11070765/s1, Table S1: (a) Cerebrovascular autoregulation and its relation to presence of IVH on cUS one week after birth, (b) Cerebrovascular autoregulation and its relation to presence of PVL on cUS one week after birth; Table S2: The clinical determinants of cerebral injury on cUS, determined by univariable logistic regression analysis; Table S3: The clinical determinants of cerebral injury on cUS, determined by multivariable logistic regression analysis.
