# Supplementary material for: Cerebrovascular Autoregulation in Preterm Infants Using Heart Rate or Blood Pressure: A Pilot Study
Source: Children (Basel). 2024 Jun 24;11(7):765. doi: 10.3390/children11070765 (PMC11276379; doi:10.3390/children11070765)
Supplement: Supplementary file 1 [file children-11-00765-s001.zip › children-3034797-supplementary.pdf]

Table S1a. Cerebrovascular autoregulation and its relation to presence of IVH on cUS one week after birth.

Day	Measurement variable	No IVH on cUS (*n* = 7)	IVH on cUS (*n* = 25)	*p*-value
1		(*n* = 7)	(*n* = 25)	
	Mean COx	0.09 + 0.08	0.07 + 0.12	0.574
	Mean TOHRx	-0.01 + 0.09	-0.04 + 0.12	0.453
	%TimeCAR with MABP (%)	28.1 + 10.9	25.6 + 13.9	0.623
	%TimeCAR with HR (%)			
	TOHRx cc cut-off 0.3	14.8 + 7.0	12.2 + 8.7	0.437
	TOHRx cc cut-off -0.3	16.1 + 11.2	18.2 + 13.8	0.690
2		(*n* = 5)	(*n* = 19)	
	Mean COx	0.04 + 0.16	0.06 + 0.12	0.853
	Mean TOHRx	0.02 + -0.49	-0.07 + 0.11	0.016*
	%TimeCARi with MABP (%)	23.0 + 12.5	25.6 + 12.3	0.690
	%TimeCARi with HR (%)			
	TOHRx cc cut-off 0.3	17.4 + 8.1	11.2 + 8.1	0.178
	TOHRx cc cut-off -0.3	11.4 + 7.6	21.0 + 12.3	0.055
3		(*n* = 5)	(*n* = 23)	
	Mean COx	-0.01 + 0.18	0.03 + 0.15	0.695
	Mean TOHRx	0.04 + 0.08	-0.03 + 0.15	0.179
	%TimeCARi with MABP (%)	21.3 + 15.4	23.8 + 12.8	0.740
	%TimeCARi with HR (%)			
	TOHRx cc cut-off 0.3	15.6 + 11.1	14.5 + 11.1	0.841
	TOHRx cc cut-off -0.3	11.9 + 5.4	18.0 + 10.6	0.089

Table S1b. Cerebrovascular autoregulation and its relation to presence of PVL on cUS one week after birth.

Day		Measurement variable	No PVL on cUS (*n* = 20)	PVL on cUS (*n* = 10)	*p*-value
1			(*n* = 20)	(*n* = 10)	
		Mean COx	0.092 + 0.097	0.044 + 0.145	0.280
		Mean TOHRx	-0.030 + 0.102	-0.026 + 0.136	0.919
		%TimeCARi with MABP (%)	27.3 + 12.5	23.6 + 14.8	0.496
		%TimeCARi with HR (%)			
		TOHRx cc cut-off 0.3	12.2 + 8.3	14.1 + 8.6	0.554

		TOHRx cc cut-off -0.3	17.3 ± 12.3	18.5 ± 15.3	0.812
2			(*n* = 15)	(*n* = 9)	
		Mean COx	0.047 ± 0.104	0.065 ± 0.154	0.723
		Mean TOHRx	-0.044 ± 0.096	-0.064 ± 0.126	0.655
		%TimeCARi with MABP (%)	26.1 ± 12.1	23.5 ± 12.8	0.621
		%TimeCARi with HR (%)			
		TOHRx cc cut-off 0.3	12.8 ± 8.3	11.8 ± 9.0	0.782
		TOHRx cc cut-off -0.3	17.1 ± 9.8	22.1 ± 15.0	0.327
3			(*n* = 19)	(*n* = 9)	
		Mean COx	0.031 ± 0.155	-0.003 ± 0.146	0.575
		Mean TOHRx	0.004 ± 0.114	-0.051 ± 0.095	0.198
		%TimeCARi with MABP (%)	24.2 ± 13.5	21.6 ± 12.6	0.631
		%TimeCARi with HR (%)			
		TOHRx cc cut-off 0.3	16.0 ± 12.1	11.9 ± 7.9	0.287
		TOHRx cc cut-off -0.3	15.3 ± 9.8	20.1 ± 10.3	0.650

Data are presented as mean ± standard deviation. IVH, germinal matrix-intraventricular hemorrhage according to Papile (modified) [22, 23]; PVL, periventricular leukomalacia according to de Vries et al [24]; cUS, cranial ultrasonography; COx, cerebral oximetry index; TOHRx, tissue oxygenation heart rate reactivity index; %timeCARi, percentage of time with impaired cerebrovascular autoregulation; MABP, mean arterial blood pressure; HR, heart rate; cc, correlation coefficient. Numbers vary per day since CAR measurements were not available for all neonates on all days in this study. * *P*-value < 0.05.

Table S2. The clinical determinants of cerebral injury on cUS, determined by univariable logistic regression analysis.

				**Univariable analysis**					
Variable	B	OR (95% CI)	*p*-value	B	OR (95% CI)	*p*-value	B	OR (95% CI)	*p*-value
Gestational age (weeks + days)				0.0	1.0 (0.9-1.1)	0.991			
Apgar 5min				0.0	1.0 (0.7-1.6)	0.891			
SGA				-0.6	0.6 (0.7-4.6)	0.585			
HsPDA				0.3	1.4 (0.3-5.9)	0.648			
NEC				-0.3	0.8 (0.1-4.1)	0.741			
EONS				20.8	1.1 x 10^9 (0.0)	0.999			
	Day 1			Day 2			Day 3		
Sedatives	-0.4	0.7 (0.1-3.2)	0.613	0.7	1.1 (0.2-4.9)	0.923	-0.7	0.5 (0.1-2.5)	0.403
Inotropes	20.7	1.0 x 10^9 (0.0)	1.00	0.7	1.9 (0.2-21.1)	0.586	20.8	1.1 x 10^9 (0.0)	0.999
Mean PCO_2	-0.1	1.0 (0.5-1.8)	0.880	-0.3	0.8 (0.3-1.6)	0.756	0.4	1.5 (0.6-3.5)	0.399
Mechanical ventilation	0.3	1.3 (0.3-5.7)	0.706	1.3	3.7 (0.8-16.8)	0.089*	2.2	9.3 (1.6-54.8)	0.014*
COx	-1.8	0.2 (0.0-101.6)	0.576	4.3	72.9 (0.0-255195.2)	0.303	-3.0	0.1 (0.0-12.9)	0.287
TOHRx	-0.4	0.7 (0.0-481.8)	0.911	-7.5	0.0 (0.0-12.1)	0.141	-1.2	0.3 (0.0-384.0)	0.740
%TimeCARi with MABP (%)	0.0	1.0 (0.9-1.1)	0.765	0.0	1.0 (1.0-1.1)	0.350	0.0	1.0 (0.9-1.0)	0.320
%TimeCARi with HR, cc cut-off 0.3 (%)	0.0	1.0 (0.9-1.1)	0.618	0.0	1.0 (0.9-1.1)	0.406	0.0	1.0 (0.9-1.1)	0.721

%TimeCARi with HR, cc cut-off -0.3 (%)	0.0	1.0 (0.9-1.1)	0.879		0.1	1.1 (1.0-1.2)	0.141		0.0	1.0 (0.9-1.1)	0.924

CUS, cranial ultrasonography; B, unstandardized coefficient; OR; odds ratio, CI, confidence interval; SGA, small for gestational age; HsPDA; hemodynamically significant patent ductus arteriosus; NEC, necrotizing enterocolitis; EONS, early onset of neonatal sepsis confirmed with a positive blood cult; PCO_2, partial pressure of carbon dioxide; COx; cerebral oximetry index; TOHRx; tissue oxygenation hear rate reactivity index; %timeCARi, percentage of time with impaired cerebrovascular autoregulation; MABP, mean arterial blood pressure; HR, heart rate; cc, correlation coefficient. * P-value < 0.10.

Table S3. The clinical determinants of cerebral injury on cUS, determined by multivariable logistic regression analysis.

**Multivariable analysis**			
Variable	B	OR (95% CI)	*p*-value
Mechanical ventilation day 2	0.1	1.1 (0.2-8.1)	0.929
Mechanical ventilation day 3	2.2	8.8 (1.0-76.6)	0.050

CUS, cranial ultrasonography; B, unstandardized coefficient; OR; odds ratio, CI, confidence interval.
